# Relationship between upper-body strength and power and shooting accuracy: a comparison of non-disabled and wheelchair basketball athletes

**DOI:** 10.3389/fspor.2026.1768525

**Published:** 2026-02-05

**Authors:** Yang Yang, Quincy R. Johnson, Angeleau A. Scott, Dimitrije Cabarkapa, Andrew C. Fry

**Affiliations:** 1 Jayhawk Athletic Performance Laboratory - Wu Tsai Human Performance Alliance, Department of Health, Sport, and Exercise Sciences, University of Kansas, Lawrence, KS, United States; 2 Fry Sports Performance, Lawrence, KS, United States

**Keywords:** accuracy, paralympics, power, sports performance, strength

## Abstract

**Introduction:**

Although wheelchair basketball (WCB) is one of the most popular Paralympic sports, limited research has focused on sports performance and strength and conditioning within the sport. The purpose of the present investigation is to examine the relationship between upper-body muscular strength, power, and shooting accuracy in WCB athletes and their non-disabled recreational basketball players participating in WCB.

**Methods:**

Twenty athletes participated in the present study, of whom ten were WCB athletes and ten were healthy college-aged recreational basketball athletes. Upper-body muscular strength and power were evaluated using bilateral handgrip strength (HGS) and the Seated Medicine Ball Throw Test (SMBT). Shooting accuracy was assessed through ten standardized 2-point field-goal attempts taken from a 5.10-meter distance. Pearson's or Spearman's correlation coefficients were used to examine associations between variables, depending on data normality, while independent t-tests were used to examine between-group differences.

**Results:**

Significantly strong positive correlations were observed between HGS, SMBT distance, and shooting accuracy in WCB athletes (*ρ* = 0.73–0.85, *p* ≤ 0.02), while non-WCB athletes displayed weak nonsignificant correlations (r ≤ 0.30, *p* ≥ 0.40). Between-group comparisons revealed significantly lower HGS in WCB athletes (*p* < 0.02, g > 0.8), while no differences were found for SMBT or shooting accuracy.

**Conclusion:**

While strength contributes to shooting performance, the findings of this study indicate that it is not the sole determinant of success. Non-disabled participants did not perform better in shooting accuracy than WCB athletes, despite the biomechanical advantage conferred by higher stature, underscoring the importance of sport-specific skill and coordination. Ultimately, shooting performance depends on integrating refined technique, experience, and strength.

## 1 Introduction

Wheelchair basketball (WCB) is among the most popular Paralympic sports, specifically designed for individuals with physical disabilities. While the game adheres to the general rules of traditional 5 × 5 basketball, certain modifications are introduced to ensure inclusivity and fairness. Central to this effort is the functional classification system, which assigns players a point value from 1.0 to 4.5, based on the extent to which their impairment limits functional trunk control and lower-limb contribution during basketball-specific tasks (1). Athletes classified as 1.0 to 1.5 exhibit minimal active trunk movement and rely primarily on upper-extremity function, whereas those classified as 4.0 to 4.5 demonstrate near complete trunk control, often including athletes with unilateral lower-limb amputations or minimal neurological impairment. During competition, the sum of the five players' classification values may not exceed 14 points, thereby ensuring balanced team composition. These functional abilities directly influence essential basketball skills such as pushing, pivoting, shooting, rebounding, dribbling, passing, and catching, which are recognized as fundamental determinants of WCB performance (1, 2).

Similar to non-disabled basketball, WCB is a high-intensity fast-paced team sport in which outcomes are heavily influenced by athletes' physical fitness, characteristics include strength, speed, endurance, and technical proficiency (3, 4). However, unlike their non-disabled counterparts, WCB athletes often face limitations in generating force from the lower-body due to congenital conditions or acquired injuries. As a result, upper-body strength and power represent key determinants of performance and have become a primary focus for both player development and sport-specific research.

Building on the critical role of the upper-body in WCB, strength and power are widely recognized as two of the most important physical traits for basketball performance (5–7). In WCB, where nearly all fundamental skills rely on the upper extremities, upper-body strength becomes particularly decisive. Key joint actions such as shoulder and elbow flexion are essential for generating the force needed to propel the ball toward the basket and ultimately determine shooting success (8). Also, these movements have been shown to differentiate proficient from non-proficient shooters in both WCB and non-disabled populations (9–12). However, despite the recognized importance of strength and power, recent findings indicate that traditional measures such as the barbell bench press and back squat one-repetition maximum do not directly predict shooting performance in non-disabled basketball players (13), suggesting that strength may function more as a foundational prerequisite than as a direct determinant of skill execution.

Handgrip strength (HGS) testing has become one of the most widely adopted methods for assessing muscular strength due to its low cost, simplicity, and practicality (14–16). In non-disabled athletes, HGS has demonstrated moderate to strong associations with sport-specific performance outcomes, including basketball shooting accuracy and handball throwing velocity (17, 18). Evidence from WCB athletes similarly highlights the role of upper-body strength, showing positive relationships with sprint performance (19). However, its connection to shooting accuracy in WCB remains less consistent, potentially due to the confounding effects of fatigue protocols incorporated into certain testing procedures (19). Furthermore, although no significant differences in HGS have been observed across functional classification levels, the technical demands of WCB strongly suggest that hand grip strength remains a critical component for executing fundamental skills such as throwing, passing, and shooting (20).

Beyond strength, anaerobic power represents another critical determinant of basketball performance (21, 22). In non-disabled populations, research has traditionally emphasized lower-body assessments using force-plate technology to evaluate neuromuscular function and power output (6, 23–26). However, this approach is less applicable to many WCB athletes, who are unable to generate substantial lower-body force due to their impairments. As a result, the upper-body Wingate Anaerobic Test has become the most widely used method of assessing anaerobic power in this population, with evidence supporting its validity and reliability (27). Notably, Wingate-derived power outputs have been shown to have significant associations with shooting performance, regardless of the functional classification level (19).

Yet, despite its usefulness, the Wingate test is time-intensive, and physically demanding, which limits its feasibility in applied sport settings. For this reason, the Seated Medicine Ball Throw (SMBT) has been recommended as a more practical field-based alternative (28). The SMBT has demonstrated strong alignment with countermovement vertical jump power indices, providing a quick and non-invasive means of evaluating upper-body power in WCB athletes (27, 28). Recent findings further highlight its value, showing moderate to strong positive correlations (r = 0.49–0.79) between SMBT performance and mid to long-distance shooting accuracy, underscoring its potential as a sport-specific and accessible assessment tool (29).

While preliminary research has investigated the relationship between shooting accuracy and muscular strength and power within WCB, the current literature on this topic is scarce, particularly for adult WCB athletes (19, 22, 29). Muscular strength and power are two of the most important physical traits for WCB athletes. By understanding this critical relationship between muscular strength and power and WCB athletic performance characteristics, such as shooting accuracy, practitioners and WCB athletes themselves may benefit from the information, specifically a proper training program to ensure optimal athletic development, and most importantly, improve performance during competition. Finally, comparing non-disabled athletes and WCB athletes may provide additional insight by isolating the effects of disability on athletic performance, such as the difference mentioned by Hanks & Gretchen (30) regarding lower-body muscle activation differences between WCB and non-disabled athletes. Therefore, the purpose of the present study was twofold: i) to examine the differences between WCB athletes and their non-disabled peers in upper-body strength and power and shooting accuracy, and ii) to examine the relationship between upper-body strength and power and shooting accuracy in both WCB athletes and their non-disabled counterparts who are participating in WCB. Based on aforementioned scientific literature, it was hypothesized that both non-disabled and WCB athletes would show a positive association between strength and power variables and shooting accuracy, with non-disabled athletes demonstrating superior overall strength and power capacities.

## 2 Methods

### 2.1 Subjects

A total of twenty male athletes volunteered to participate in this investigation. The first group consisted of ten professional WCB players from a National Wheelchair Basketball Association (NWBA) Division-I team (age = 33.2 ± 8.3 years; sitting height = 119.7 ± 9.3 cm; body mass = 77.6 ± 13.8 kg). All WCB athletes have at least 3 years of continuous playing experience. The second group consisted of ten college-aged non-wheelchair-bound participants who were all recreational basketball players with at least 5 years of playing experience and regularly participated in resistance training weekly for at least 3 years (age = 24.3 ± 3.1 years; sitting height = 137.6 ± 5.0 cm; body mass = 93.8 ± 17.8 kg). All participants were free from upper-body musculoskeletal injuries that could limit or impair their participation in the testing procedures. The testing procedures performed in the present study were approved by the Institutional Review Board (STUDY00151547), and all participants provided informed consent prior to data collection. Given the limited sample size and exclusion of female participants, the present investigation was conducted as an exploratory study to identify preliminary relationships rather than to establish definitive causal inferences. The sample size was determined by participant availability and feasibility within a professional WCB setting and was intended solely to support exploratory analyses.

### 2.2 Procedures

#### 2.2.1 Anthropometrics

Sitting height was measured using a portable stadiometer (SECA 213, SECA, Hamburg, Germany) while participants remained seated in their wheelchairs. Body mass was assessed with a wheelchair scale (Angel USA, NWC series, USA). Participants were first weighed while seated in their wheelchairs, after which the wheelchair weight was measured separately and subtracted from the total to calculate individual body mass.

#### 2.2.2 Muscular strength

Upper-extremity muscular strength was assessed using the handgrip strength test which was measured with a hydraulic hand dynamometer and served as the primary analytical measurement of maximal upper-extremity muscular strength (Baseline Evaluation Instruments, Fabrication Enterprises Inc., New York, USA). Hand lengths were measured from the styloid process of the ulna to the top of the middle finger via digital calipers (Neiko 01409A 12” Electronic Digital Caliper, China) (31). The dynamometer's handle was adjusted according to the participants' hand length (32). Participants began each trial with the elbow positioned at approximately 90° of flexion. While seated upright in their wheelchairs, participants performed the test on the researchers' command (e.g., “3-2-1-go”) by pulling the handles together with maximal isometric effort for 3 s. Each participant completed three trials with 30 s of rest between attempts. For analysis purposes, the best trial from both the dominant and non-dominant hands was used, defined as the highest recorded force (kg). Dominant hand was determined by the participant's preferred shooting hand.

#### 2.2.3 Muscular power

Upper-extremity anaerobic power was assessed using the SMBT, a test shown to be reliable for evaluating explosive power (33, 34). Participants positioned their wheelchairs at the starting line, ensuring the front bar did not cross it. A research assistant anchored the wheelchair to prevent movement during the test. On the researcher's verbal command (e.g., “3-2-1-go”), participants executed a chest pass with a 5 kg medicine ball, throwing it forward with maximal effort. The distance from the front bar of the wheelchair to the ball's first point of ground contact was measured in centimeters. Each participant completed three throws with a 60–90 s rest between each trial, and the best trial (i.e., greatest distance) was used for performance analysis purposes.

#### 2.2.4 Shooting accuracy

Shooting accuracy was assessed through ten two-point shot attempts, each recorded as either a make or a miss. All shots were taken while directly facing the basket from a standardized distance of 5.10 m. Participants aligned the front bar of their wheelchairs with tape on the floor to ensure consistent positioning during roll-in shot attempts. A research assistant was present to assist with rebounding and passing to minimize distractions. The basketball hoop height (3.05 m) and rim diameter (0.75 m) conformed to NWBA regulation standards.

### 2.3 Statistical analysis

The Shapiro–Wilk test was used to assess normality. As the WCB group data violated this assumption, descriptive statistics for this group are reported as medians with interquartile ranges. For the non-WCB group, which met the normality assumption, descriptive statistics are presented as means with standard deviations. To test associations between variables, Spearman's rank correlation was used for data that violated the normality assumption (*ρ* = 0.10–0.40 weak, *ρ* = 0.40–0.60 moderate, *ρ* > 0.60 strong), while Pearson's correlation was applied to data that met the normality assumption (r = 0.10–0.30 weak, r = 0.31–0.50 moderate, r > 0.50 strong).

To examine within-group differences, a Wilcoxon signed-rank test was used to compare maximal handgrip strength between dominant and non-dominant hands in the WCB group. At the same time, a paired t-test was applied for the non-WCB group. To assess between-group differences, independent t-tests were conducted for each variable examined in the study. Statistical significance was set *α* priori to *p* < 0.05. Based on the sample size (*n* = 20), Hedge's g was used to calculate the measure of effect size (i.e., g = 0.2–0.49 small, g = 0.5–0.80 moderate, and g > 0.8 large) (35). All statistical analyses were completed with SPSS (Version 29.0; IBM Corp., Armonk, NY, USA) and R Studio (RStudio 2025.09.0, Boston, MA).

## 3 Results

Descriptive statistics were calculated for each dependent variable and are presented in Table 1 as means and standard deviations (¯X ± SD) or medians and interquartile ranges [M (IQR)]. The non-WCB group demonstrated significantly greater HGS in both the dominant and non-dominant hands compared to the WCB group (*p* < 0.020). Across both groups, dominant and non-dominant-HGS were strongly and positively correlated (WCB: *ρ* = 0.942, *p* < 0.001; non-WCB: r = 0.929, *p* < 0.001). Within-group comparisons revealed no significant difference between dominant and non-dominant-hand strength in the WCB group (*p* = 0.836), whereas the non-WCB group exhibited significantly greater strength in the dominant hand (*p* = 0.007).

**Table 1:** Descriptive statistics, mean and standard deviation (x¯ ± SD) or median and interquartile range [M(IQR)], *p*-value, and effect size for wheelchair basketball (WCB) athletes and non-disabled recreational basketball athletes.

Metric	WCB	Non-WCB	*p*-value	Effect size
Age (yrs)	33.2 ± 8.3^a^	24.3 ± 3.1	0.005	−1.36
Sitting Height (cm)	119.7 ± 9.3^a^	137.6 ± 5.0	0.001	2.29
Body Mass (kg)	77.6 ± 13.8^a^	93.8 ± 17.8	0.017	0.978
Handgrip Strength – D (kg)	53.0 [16.0]^a^	65.0 ± 9.3	0.003	1.313
Handgrip Strength – ND (kg)	51.0 [15.3]^a^	60.6 ± 10.6	0.030	0.861
SMBT distance (m) – 5kg	4.28 [0.71]	4.47 ± 0.68	0.220	0.339
Shooting Accuracy (%)	20.0 [45.0]	29.00 ± 17.3	0.461	0.042

D, Dominant; ND, Non-dominant; SMBT, seated medicine ball throw test.

^a^ denotes significant difference compared to the Non-WCB group.

In the WCB group, handgrip strength was strongly and positively associated with shooting accuracy (dominant hand: *ρ* = 0.733, *p* = 0.016; non-dominant hand: *ρ* = 0.789, *p* = 0.007). In contrast, the non-WCB group displayed weak and non-significant negative associations (dominant hand: r = –0.228, *p* = 0.526; non-dominant hand: r = –0.178, *p* = 0.623).

**Figure 1:**
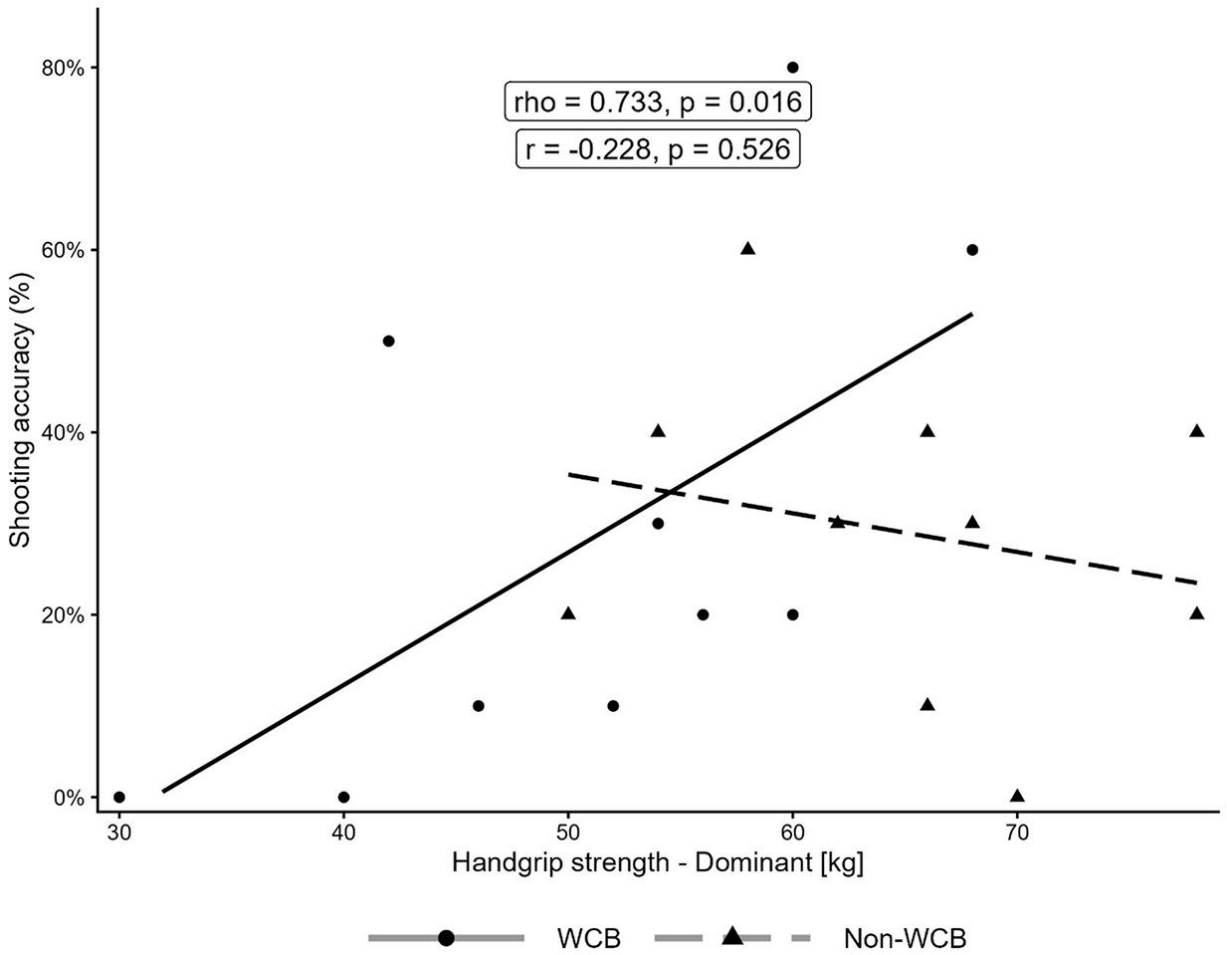
Relationship between dominant handgrip strength and two-point shooting accuracy. The solid line represents the regression line for the WCB group, and the dashed line represents the regression line for the non-WCB group. For correlation coefficients, the WCB group was assessed with Spearman's rho, and the non-WCB group was assessed with Pearson's r.

**Figure 2:**
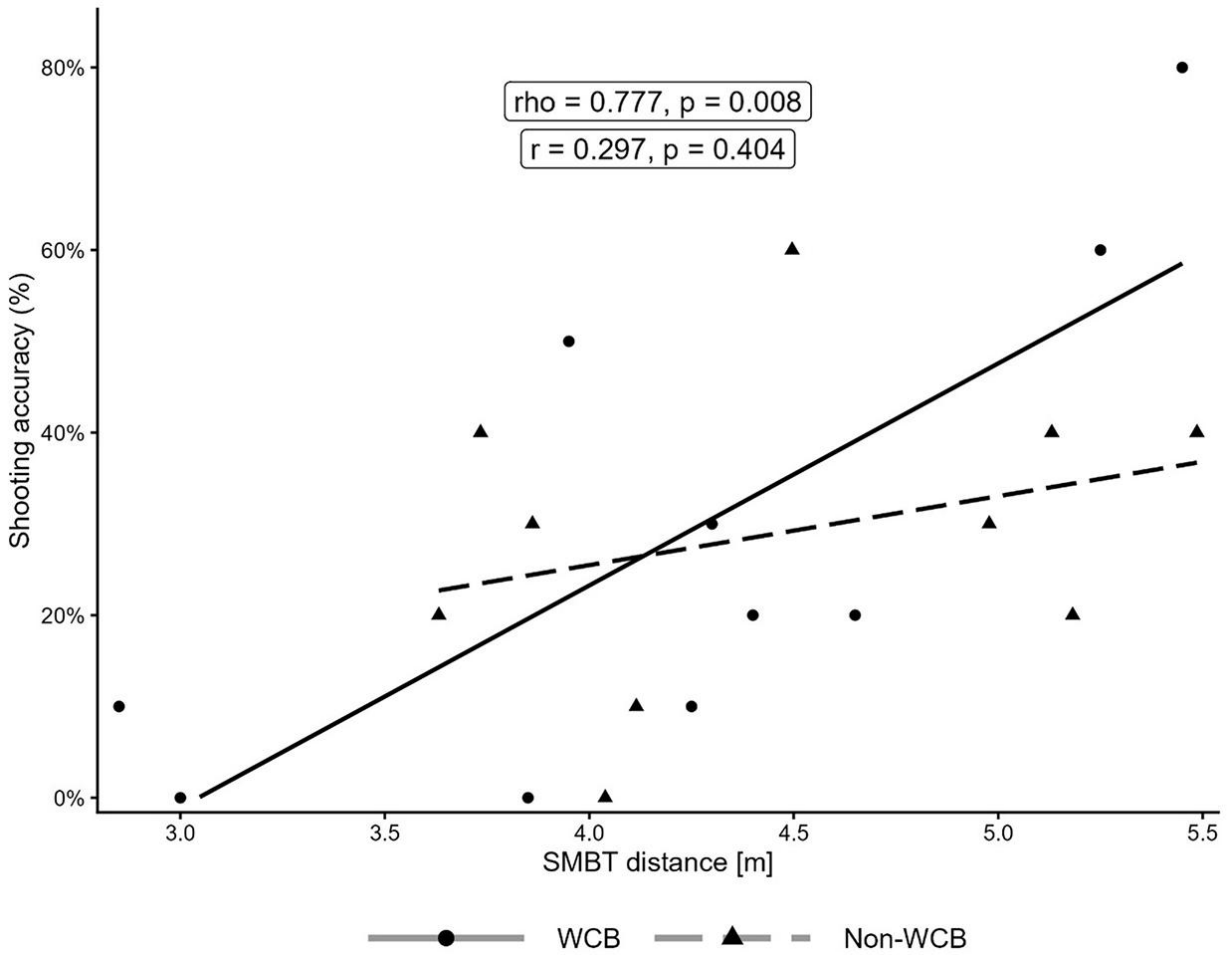
Relationship between non-dominant handgrip strength and two-point shooting accuracy. The solid line represents the regression line for the WCB group, and the dashed line represents the regression line for the non-WCB group. For correlation coefficients, the WCB group was assessed with Spearman's rho, and the non-WCB group was assessed with Pearson's r.

**Figure 3:**
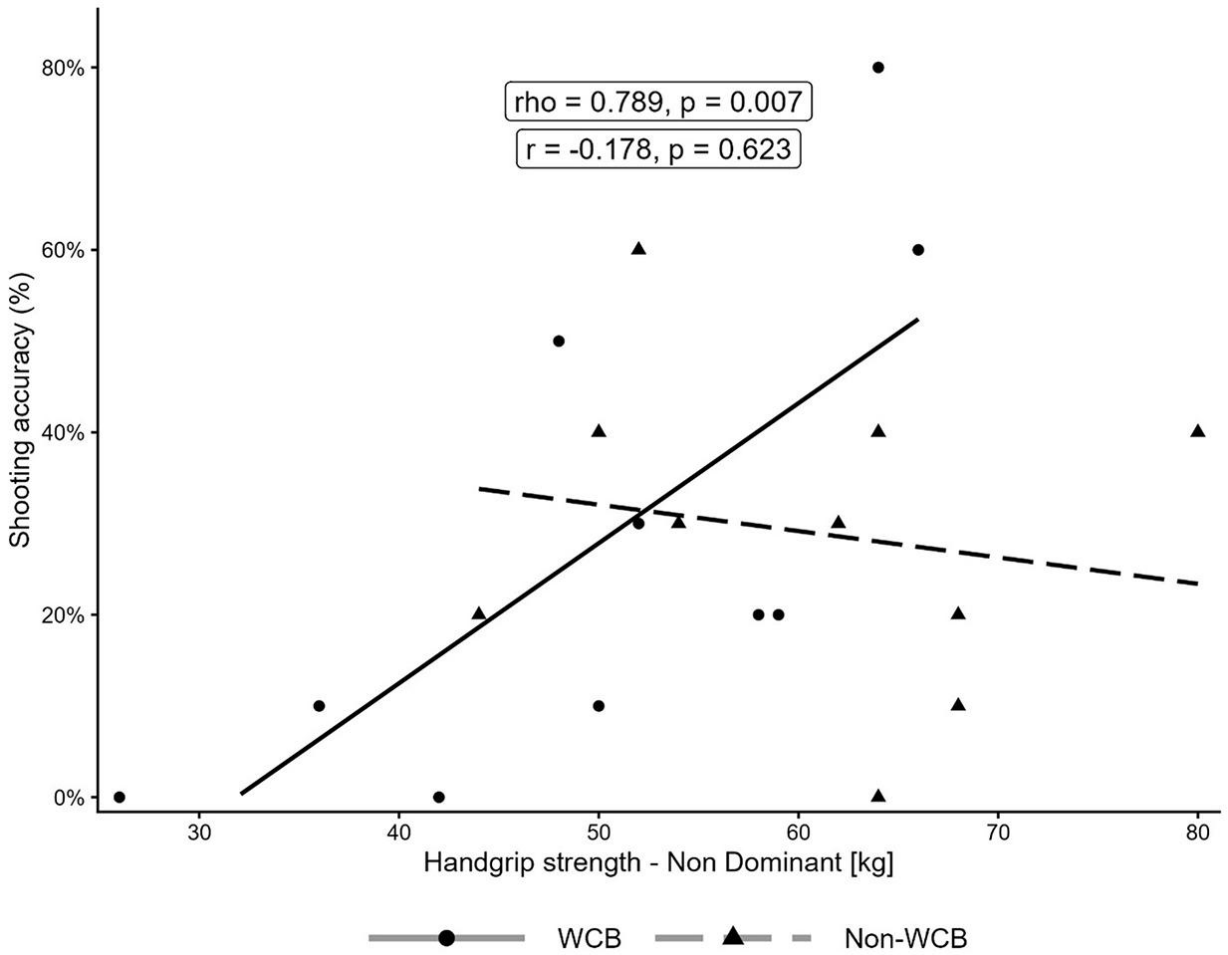
Relationship between SMBT distance and two-point shooting accuracy, and two-point shooting accuracy. The solid line represents the regression line for the WCB group, and the dashed line represents the regression line for the non-WCB group. For correlation coefficients, the WCB group was assessed with Spearman's rho, and the non-WCB group was assessed with Pearson's r.

Similarly, SMBT distance was strongly and positively correlated with shooting accuracy in the WCB group (*ρ* = 0.777, *p* = 0.008) but was only weak and non-significant in the non-WCB group (r = 0.297, *p* = 0.404). Importantly, HGS in both hands showed strong, significant positive correlations with SMBT distance in both groups (WCB: *ρ* = 0.845, *p* = 0.002 [dominant], *ρ* = 0.794, *p* = 0.006 [non-dominant]; non-WCB: r = 0.706, *p* = 0.022 [dominant], r = 0.672, *p* = 0.033 [non-dominant]). All correlations between the variables are illustrated in Figures 1–3.

## 4 Discussion

The purpose of the present investigation was to examine differences in upper-body muscular strength and power between WCB athletes and their non-WCB counterparts, as well as to explore the relationships between these variables and mid-range shooting accuracy. To the best of our knowledge, this is the first study to assess the associations between HGS, upper-body power, and shooting performance within a cohort of professional WCB athletes. Although both dominant and non-dominant handgrip strength values were significantly greater in the non-WCB group compared to WCB athletes (*p* ≤ 0.030), strong positive correlations between handgrip strength and shooting accuracy were observed only among WCB athletes (*ρ*≥ 0.733). Conversely, the non-WCB group exhibited weak negative correlations between bilateral handgrip strength and shooting accuracy (r = –0.228–0.178). Similarly, while SMBT distance was positively associated with shooting accuracy in both groups, this relationship was markedly stronger in WCB athletes (*ρ* ≥ 0.777) than in their non-WCB counterparts (r = 0.297). Overall, these findings suggest that in the absence of lower-body involvement, WCB athletes rely more heavily on upper-body force generation and control. Their proficiency likely reflects a high level of skill adaptation, enabling them to optimize upper-body coordination to compensate for the lack of lower-body contribution. In contrast, non-disabled recreational basketball athletes typically integrate lower-body movement and kinetic chain dynamics when shooting; however, when these elements are removed, as in a seated position, the movement becomes biomechanically unfamiliar, rendering it a distinct skill for which strength alone is insufficient to sustain accuracy. Interpretation of between-group comparisons should account for substantial differences in age, sitting height, body mass, and strength between groups. These discrepancies reflect the practical challenges of recruiting closely matched non-disabled basketball athletes to compete against elite wheelchair basketball athletes and indicate that the groups were not intended to be equivalent beyond the seated-task constraint. Accordingly, between-group findings should be interpreted descriptively, with greater emphasis placed on within-group associations that reflect task-specific adaptation.

Previous research has demonstrated mixed findings regarding the association between HGS and shooting performance in non-disabled basketball athletes (36, 37). For example, Vadillo-Ventura et al. (36) reported a strong positive association between HGS and 3-point shooting accuracy among a cohort of international and national-level basketball athletes aged 12–18 years, which is similar to the results obtained in the present investigation, where strong positive association was found between both dominant and non-dominant HGS and shooting accuracy within the WCB group. On the other hand, when studying a cohort of youth basketball athletes (13–14 years old), Apostolidis & Emmanouil (37) reported no significant association between HGS and basketball shooting performance, which is contradictory to the aforementioned findings, including the ones observed in this study. A possible explanation for these differing conclusions may relate to the age differences between the cohorts examined. Specifically, youth basketball athletes, such as those studied by Apostolidis and Emmanouil (37), may not yet possess sufficient basketball shooting experience or strength at 13–14 years of age, in contrast to the participants in the present investigation, who despite being WCB athletes, were all adults. In addition, prior research on WCB athletes has explored the relationship between shooting performance, HGS, and upper-body power using the upper-body Wingate Anaerobic Test, indicating that upper-body muscular power may be more strongly associated with shooting performance than grip strength (19). This observation aligns with the results of the present investigation, which demonstrated a strong association between SMBT distance and mid-range shooting accuracy (i.e., 2-point shots). However, Soylu et al. (19) did not detect a significant relationship between HGS and shooting performance, whereas a strong association between these variables was observed in the present study. This discrepancy may be attributed to methodological differences, as Soylu et al. (19) employed a zone-shot test that likely induced fatigue, while the current investigation utilized a mid-range stationary shooting protocol conducted under non-fatigued conditions. Given that fatigue has been shown to influence shooting accuracy negatively (38), these methodological distinctions may help explain the contrasting results between studies. Moreover, another important factor to consider is the difference in competition level, as WCB athletes competing at higher levels may possess greater overall strength, a trend also evident in non-disabled basketball populations (39).

While strength appears to play an important role in overall physical capacity, it is unlikely to be the sole determinant of shooting performance. In line with this notion, recent evidence suggests that muscular strength alone may not differentiate shooting proficiency among well-trained athletes. For instance, in a recently published study, Cabarkapa et al. (13) reported no significant association between upper-body and lower-body strength and free-throw, 2-point, and 3-point shooting accuracy within a cohort of well-trained male and female basketball players. The authors further proposed that these athletes likely possessed sufficient baseline strength due to their resistance training background, diminishing the influence of strength on skill-dependent outcomes. Similarly, in the present investigation, all participants, regardless of testing group, exhibited HGS values among the highest reported in WCB athletes competing on a professional level of play (19, 20, 22). Additionally, despite the non-WCB group demonstrating superior grip strength compared to the WCB group, no corresponding improvement in shooting accuracy was observed. Overall, these findings reinforce the notion that greater strength does not necessarily translate to enhanced basketball shooting performance and consistent with previous observations in non-disabled basketball, it appears that the skill component may play a more decisive role (13). In addition, it should be noted that kinematic variables such as elbow flexion, release angle, elbow height, and forearm alignment are critical technical determinants of shooting performance (40–42). While lower-body kinematic variables may have limited relevance in WCB athletes, upper-body mechanics, given their predominant contribution to propulsion and shot control, may serve as valuable coaching cues for optimizing shooting technique. Supporting this, a recent review highlighted that trunk lean, wrist flexion, shoulder flexion, elbow flexion, and release height are strongly associated with successful free-throw attempts (12), emphasizing the central role of upper-body kinematics in enhancing shooting performance among WCB athletes.

Biomechanical research in non-disabled basketball consistently demonstrates that taller athletes possess measurable advantages during shooting tasks, largely due to more favorable release mechanics (43). Although sitting height was not a primary focus of the present investigation, the WCB athletes were markedly shorter than their non-disabled counterparts. However, this anticipated advantage (i.e., greater overall height), did not result in superior shooting accuracy among the non-disabled participants. This outcome contrasts with previous findings in non-disabled basketball, where taller athletes typically benefit from a higher release point that permits lower release angles and velocities because the ball must travel a shorter vertical distance (44–46). Taken together, these findings suggest that while physical characteristics establish an important base for shooting performance in WCB, they are not sufficient to meaningfully differentiate accuracy outcomes. Continued improvement in shooting proficiency likely depends more on sport-specific skill development and the refinement of technical execution. Nevertheless, practitioners working with WCB athletes should design resistance-training programs that target upper-extremity muscular strength and power (e.g., medicine ball throw, bench press) to develop foundational prerequisites for proper skill and technical development.

While the present study offers deeper insight into the determinants of WCB shooting performance and demonstrates that strength is not the sole contributing factor, several limitations should be acknowledged. First, the relatively small sample size may have influenced the statistical power of the findings. However, considering the limited roster sizes typical of WCB teams (n ≈ 12), the results remain meaningful and informative. Second, not all key anthropometric measures were taken that may influence WCB shooting biomechanics (e.g., wingspan) (3). Additionally, the present study did not directly quantify the mechanical force or impulse requirements associated with shooting from a seated position compared to shooting while standing. This limited the ability to distinguish how wheelchair-specific constraints may alter force-generation strategies during shot execution. Finally, all shooting assessments were conducted in a controlled laboratory environment under non-fatigued conditions, which may not accurately replicate the dynamic and competitive nature of actual WCB gameplay, as the presence of fatigue may change the shooting mechanics (38). Thus, future research should focus on exploring the relationship between WCB shooting performance, upper-body kinematic patterns, and resistance-training experience. Moreover, investigations spanning different genders, competition levels (e.g., youth, collegiate, and professional divisions), and player classifications (e.g., 1.0 vs. 4.5) are warranted to provide a more comprehensive understanding of how technical, physical, and contextual factors collectively influence shooting performance in WCB.

### 4.1 Conclusion

The findings of the present investigation suggest that upper-body strength and power are strongly associated with shooting accuracy among WCB athletes, but not among non-disabled recreational basketball athletes participating in WCB. Although both dominant and non-dominant handgrip strength values were significantly greater in the non-WCB group, strong positive correlations between handgrip strength and shooting accuracy were observed only among WCB athletes. Similarly, while SMBT distance was positively related to shooting accuracy in both groups, the relationship was markedly stronger in WCB athletes, suggesting that in the absence of lower-body involvement, they rely more heavily on upper-body force generation and control. This likely reflects sport-specific skill adaptation that enables WCB athletes to optimize upper-body coordination to compensate for the lack of lower-body contribution, emphasizing that strength and power alone are not sufficient to determine shooting performance; instead, they serve as the foundation for WCB shooting performance. However, given the relatively small sample size, these findings should be interpreted with caution and viewed as preliminary. Collectively, the results indicate that upper-body strength and power are important foundational physical qualities that support shooting performance in WCB, rather than being sole determinants of shooting accuracy.

## Data Availability

The raw data supporting the conclusions of this article will be made available by the authors, without undue reservation.
